# Alterations in the gut microbiota and the efficacy of adjuvant probiotic therapy in liver cirrhosis

**DOI:** 10.3389/fcimb.2023.1218552

**Published:** 2023-07-07

**Authors:** Zengrong Wu, Hejun Zhou, Deliang Liu, Feihong Deng

**Affiliations:** ^1^ Department of Gastroenterology, The Second Xiangya Hospital, Central South University, Changsha, Hunan, China; ^2^ Research Center of Digestive Disease, Central South University, Changsha, Hunan, China

**Keywords:** cirrhosis, gut microbiota, gut-liver axis, short-chain fatty acids, probiotics

## Abstract

**Background:**

Liver cirrhosis is the end stage of various chronic liver diseases (CLDs). The gut microbiota can impact the liver environment and trigger chronic liver inflammation through the gut-liver axis. Alteration of the gut microbiota has become an effective strategy in the biological treatment of cirrhosis.

**Methods:**

Twenty-eight patients with liver cirrhosis and 16 healthy individuals were included, and fresh stool samples were collected. We analyzed changes in the gut microbiota between groups by 16S rRNA sequencing and evaluated the association between microbiota alterations and hepatic function. Additionally, 102 cirrhotic patients were retrospectively enrolled and divided into a probiotic group (n=44) and a nonprobiotic group (n=58) in addition to standard treatment for cirrhosis. Patients were monitored for hematological parameters and hepatic function during the six-month follow-up.

**Results:**

The gut microbiota profile of patients with cirrhosis was greatly different from that of healthy individuals, presenting with significantly reduced α diversity and decreased abundance of representative SCFA-producing bacteria including *Firmicutes*, *Coprococcus* and *Clostridium IV*. The pathogenic bacteria *Gammaproteobacteria*, *Veillonella*, and *Bacilli* were greatly enriched in cirrhotic patients. Additionally, patients with decompensated cirrhosis (DCPC) had a significantly reduced abundance of *Oscillibacter* compared to compensated cirrhosis (CPC), which is also a SCFA-producing bacteria, and the lower *Firmicutes* to *Bacteroidetes* ratio and enhanced MDR values were also shown in DCPC patients compared to CPC patients. In addition, the abundance of *Firmicutes* was negatively related to hepatic function in cirrhotic patients, including the levels of ALT, AST, and DBIL. From the retrospective study, we found that biochemical improvements in alanine transaminase (ALT) and total bilirubin (TBIL) were obtained in DCPC patients who received oral probiotic therapy compared with the nonprobiotic group.

**Conclusion:**

Severe microbial dysbiosis existed in patients with liver cirrhosis, especially patients who reached the decompensatory stage. SCFA-producing bacteria were significantly reduced in cirrhosis. Altered gut microbiota cause changes in functional modules, which may contribute to cirrhosis progression and are associated with clinical prognosis. Adjuvant probiotic supplementation to enhance SCFA-producing bacteria can be a prospective therapy for patients with cirrhosis.

## 1 Introduction

Liver cirrhosis is the end stage of various chronic liver diseases (CLDs), which generally occur after long-term chronic inflammation of the liver, where the healthy liver parenchyma is gradually replaced by fibrotic tissue and regenerative nodules, and can complicate portal hypertension (Ginès et al., 2021). Cirrhosis is currently the 11th leading cause of death worldwide; approximately 1.5 billion persons have CLD worldwide, and the incidence of CLD and cirrhosis is 20.7/100,000, posing a substantial global burden (Moon et al., 2020). The common causes of cirrhosis are chronic viral hepatitis and alcoholic liver disease. In recent years, the incidence of hepatitis virus-related chronic liver disease has decreased with the implementation of hepatitis B vaccination and antiviral treatment, but the incidence of alcoholic and fatty liver disease has increased due to changes in people’s diet and lifestyle (Moon et al., 2020). Although etiological treatment has a therapeutic effect on cirrhosis, there are still no effective antifibrotic drugs approved; thus, it remains a clinical challenge in the treatment of liver cirrhosis (Fallowfield et al., 2021).

The gut microbiota is a dynamic entity that coevolves with the host (Adak and Khan, 2019). The gut microbiome contains at least 100 times more genes than the body’s own genome (Gill et al., 2006). There are three main microorganisms in the adult gastrointestinal tract: bacteria, archaea, and eukaryotes, with bacteria accounting for the largest proportion (Bäckhed et al., 2005). Gut microbiota and their metabolites could impact host digestion, metabolism, and immunity, thus playing an important role in human health and the progression of numerous diseases (Gomaa, 2020). Liver damage is associated with small intestinal bacterial overgrowth and microbial dysbiosis of the gastrointestinal tract (Philips et al., 2017). The levels of *Veillonella*, *Streptococcus*, *Clostridium*, and *Prevotella* were increased, while *Bacteroides* was significantly decreased in the gut microbiota of patients with liver cirrhosis when compared with healthy controls (Qin et al., 2014), and alterations in bacterial composition can lead to significant changes in gene function, which may be one of the causes of liver cirrhosis (Shu et al., 2022). The gut-liver axis is the bidirectional connection between the gut and its microbiota therein and the liver, leading to the interaction of signals generated by dietary, genetic, and environmental factors. The establishment of this bidirectional connection relies on the portal vein and the biliary system (Albillos et al., 2020). Gut-derived products can reach the liver via the portal vein, and the liver releases bile acid to the intestine via the biliary tract (Lee and Suk, 2020). Balancing the gut microbiome is critical for maintaining homeostasis of the gut-liver axis. At homeostasis, the intact intestinal mucosal and vascular barrier facilitate nutrition absorption and limit the systemic dissemination of microbes and toxins to the liver. Upon microbial dysbiosis, increased pathogenic bacterial load and their products could disrupt the gut barrier and allow the bacteria and their products to cross (Tripathi et al., 2018). These microbial- (or pathogen-) associated molecular patterns (MAMPs/PAMPs) are recognized by immune receptors on the lamina propria of gut and liver cells, such as Kupffer cells and hepatic stellate cells, which initiate inflammatory cascades that ultimately lead to liver damage and fibrosis (Uesugi et al., 2001; Csak et al., 2011; Seki and Schnabl, 2012; Anand et al., 2016).

Short-chain fatty acids (SCFAs), mainly acetate, propionate, and butyrate, are the main metabolites produced in the colon by bacterial fermentation of dietary fibers (Martin-Gallausiaux et al., 2021). SCFAs are essential for gut integrity by regulating the luminal pH, providing energy for epithelial cells and affecting mucosal immune function (Blaak et al., 2020), and they exert important roles in the progression of cardiovascular diseases (Hu et al., 2022), neurodegenerative diseases (Yadav et al., 2022) and ischemic strokes (Chen et al., 2019). In cirrhotic patients, the capacity of SCFA-producing fecal microbiota is reduced (Jin et al., 2019) and linked to the development of hepatic encephalopathy (Bloom et al., 2021). Therefore, the gut microbiome and its metabolites may affect the liver microenvironment and are closely associated with the progression of liver inflammation and cirrhosis.

Since gut microbiome alterations correlated with the severity, prognosis, and several complications of cirrhosis (Solé et al., 2021), probiotic therapy, which improves the composition of gut microbiota, has been increasingly studied in patients with liver cirrhosis in recent years (Pereg et al., 2011). Probiotic-assisted therapy can reduce variceal rebleeding events in cirrhotic patients and delay the occurrence of rebleeding after endoscopic therapy (Zhang et al., 2020). In addition, probiotics are recommended as a primary treatment for patients with hepatic encephalopathy (Agrawal et al., 2012; Lunia et al., 2014), as they could markedly improve the prognostic outcome of patients presenting with decreased Child-Pugh and Model for End-Stage Liver Disease (MELD) scores (Dhiman et al., 2014). Thus, probiotic supplementation is an essential therapeutic strategy for patients with liver cirrhosis.

With this background, we designed a cross-sectional study to analyze gut microbiota alterations in patients with cirrhosis and performed a retrospective study to assess the efficacy of probiotics in patients with cirrhosis. We found a profoundly abnormal gut microbiome in cirrhosis compared with healthy subjects with a characterization of abnormalities of 16S rRNA and demonstrated that adjuvant probiotic therapy can be helpful in improving the liver function of patients with cirrhosis.

## 2 Methods

### 2.1 Population and study design

For gut microbiota analysis, 28 cirrhotic patients (8 were CPC, 20 were DCPC) and 16 age- and gender-matched healthy volunteers who were admitted to the Second Xiangya Hospital of Central South University from April 2022 to November 2022 were included. For the retrospective study, 102 cirrhotic patients with cirrhosis at the Second Xiangya Hospital of Central South University from January 2017 to November 2022 were selected. Inclusion criteria for patients with liver cirrhosis were age ≥ 18 years, and the diagnosis of cirrhosis was confirmed by liver biopsy or a combination of clinical, biochemical, ultrasound, elastographic and endoscopic examinations. Exclusion Criteria were use of gut microecological agents such as probiotics and prebiotics, antibiotics and ursodeoxycholic acid in last 3 months, previous intestinal resection and carcinoma, intestinal infectious, immune diseases, diabetes mellitus, obesity, psychosomatic disorders, organ failures, pregnant/lactating women and lack of informed consent. The study was approved by the Ethics Committee of the Second Xiangya Hospital of Central South University (number: LYF2022112), and all study subjects provided informed consent.

For the retrospective study, 102 patients with cirrhosis were subdivided into a probiotic group and a nonprobiotic group, and they all received standard treatment according to previous reports(Yoshiji et al., 2021). The probiotics used in this study is commonly used and produced in China, is “Live Combined Bifidobacterium, Lactobacillus, and Enterococcus Capsules, Oral”, and the amount of probiotics was calculated using the defined daily dose. Patients who received ≥28 days of probiotics were enrolled in the probiotics group, while patients who didn’t receive probiotics were included in the nonprobiotics group. We collected basic information about the patients, including age, sex, and etiology. Routine blood tests and liver function tests were also performed. Child-Pugh classification was used to assess hepatic dysfunction. Patients were followed up for six months.

### 2.2 Fecal sample collection and 16S rRNA analysis

Fecal samples from 28 cirrhotic patients and 16 healthy subjects were collected. DNA group samples of acceptable quality were selected, and 338F (5’-ACTCCTACGGGAGGCAGCAG-3’) and 806R (5’-GGACTACHVGGGTWTCTAAT-3’) primers were used to configure the PCR system and amplify the V3-V4 variable region. Then, the PCR amplification products were purified and dissolved in Elution Buffer (MagPure Stool DNA KF Kit B was used for DNA extraction). The qualified DNA samples tested were sequenced by the DNBSEQ platform (provided by Beijing Genomics Institution). The data were filtered to keep the reads that could match to the primers, and then use cutadapt v2.6 to remove primers and reads that are contaminated by adapter sequences, and remove the reads shorter than 75% of initial length, as well as reads with ambiguous base, reads with low complexity, to acquire clean data (qualified reads) for analysis. The qualified reads were pulled into tags. Tags were clustered to generate OTUs (operational taxonomic units) according to 97% sequence similarity, and OTU representative sequences were aligned against the database for taxonomic annotation by RDP classifer (1.9.1) (sequence identity was set to 0.6). R software was used to analyze the relative abundance and diversity. PICRUSt2 was used to predict the functional abundance of the microbiota community. α diversity was used to describe the abundance of different bacterial taxa measured in one sample and it includes the ace index, chao index, shannon index and simpson index depending on the different calculation method.βdiversity was used to describe microbial diversity in different samples. Dimensionality reduction analysis using partial least squares discriminant analysis maximizes the distance between samples in two groups and can visualize intra- and intergroup differences. In addition to species richness, the LDA (linear discriminant analysis) values were used in the analysis of species differences to determine the intergroup differences of species and the extent of their contribution, and LEfSe (LDA effect size) analysis, an analytical tool for the discovery and interpretation of biomarkers in high-dimensional data, allowing the comparison of two or more subgroups with an emphasis on statistical significance and biological relevance, and also allowing the identification of biomarkers that are statistically different between groups.

### 2.3 Analysis of short-chain fatty acid contents in fecal samples

SCFAs in fecal samples among different groups were examined using high-performance liquid chromatography (HPLC; Shimadzu, Japan). SCFA samples were prepared by homogenization of fecal samples and centrifuged at 12,000 g at 4°for 10 min. SCFAs were separated using a chromatographic column (Kinetex C18, 2.6µm 100 x 3.00mm, Phenomenex, USA) with an isocratic mobile phase (acetonitrile; Fisher, USA) set at a flow rate of 0.7 ml/min and then identified at a wavelength of 210 nm using an liquid chromatography mass spectrometer (QTRAP 5500, SCIEX, USA).

### 2.4 Statistical analysis

Statistical analysis was performed using SPSS 26.0 and R software. Normally distributed data are described as the mean ± standard deviation, and nonnormally distributed data are described as the median and interquartile spacing. Counting data are described as frequency and percentage. Comparisons of count data were made using the chi-square test, and comparisons of measurement data were made using the t test or Wilcoxon signed rank sum test, with a two-sided *p*<0.05 defined as a statistically significant difference.

## 3 Results

### 3.1 Gut microbiota analysis in patients with liver cirrhosis and healthy individuals

#### 3.1.1 General information of the study subjects

A total of 44 study subjects were recruited, including 28 cirrhotic patients and 16 healthy controls, with mean ages of 54.79 ± 12.68 and 54.25 ± 10.14 years and male to female ratios of 17/11 and 9/7, respectively; the gender and age of the two groups were matched and comparable (*p*>0.05, **Table 1**). Among the included patients with cirrhosis, 20 patients were diagnosed with decompensated cirrhosis (DCPC), and 8 patients were diagnosed with compensated cirrhosis (CPC). The liver function of patients with cirrhosis was graded according to the Child-Pugh score. Of these, 20 were identified as Child-Pugh A, 6 as Child-Pugh B, and 2 as Child-Pugh C. From the analysis of laboratory indicators, we found that the white blood cell count, hemoglobin, platelet count, and albumin were significantly decreased, while the alanine transaminase, aspartate aminotransferase, total bilirubin, indirect bilirubin, direct bilirubin, total bile acids, serum creatinine, prothrombin time, prothrombin time activity and international normalized ratio were significantly increased in patients with cirrhosis. The white blood cell count, neutrophil count, hemoglobin, and platelet count were significantly decreased in DCPC compared to CPC, while there was no difference in other liver-related parameters, including aminotransferases, bilirubin, ALB and PT (*p*>0.05, **Table 1**).

**Table 1:** The demographic information, Child-Pugh grade and laboratory indication of LC and HC.

	Indicator	Normal range	LC		HC	P (LC vs. HC)	P (CPC vs. DCPC)
			CPC	DCPC			
Demographic indicators	Male(%)		17(60.7%)		9(56%)	0.77	/
			5(62.5%)	12(60%)		/	0.952
	Age		54.79 ± 12.68		54.25 ± 10.14	0.89	/
			49.00 ± 12.84	57.10 ± 12.17		/	0.257
Child-Pugh grade	Child-Pugh A		7	13			
	Child-Pugh B		0	6	/	/	/
	Child-Pugh C		1	1			
Laboratory indicator	WBC(*10^9/L)	3.5-9.5	5.53 ± 1.23	3.29 ± 1.23*	5.20 ± 1.26	0.009	<0.001
	N(*10^9/L)	1.8-6.3	3.59(2.78,4.58)	1.68(1.35,2.41) *	3.07 ± 0.86	0.106	0.001
	N%	40.0-75.0	70.00(59.90,73.15)	64.95(49.78,72.50)	59.04 ± 7.17	0.075	0.309
	Hb(g/L)	130-175	137.25 ± 19.69	105.55 ± 26.68*	128.13 ± 6.23	0.022	0.005
	PLT(*10^9/L)	125-350	100.50(92.00,161.75)	60.50(46.50,86.75) *	179.94 ± 22.86	<0.001	0.004
	ALT(U/L)	7.0-40.0	40.30(22.58,111.93)	20.30(15.00,50.75)	19.90 ± 9.32	0.045	0.170
	AST(U/L)	13.0-35.0	32.85(27.33,92.65)	30.75(24.38,51.00)	17.38 ± 4.87	<0.001	0.629
	TBIL(μmmol/l)	3.4-17.1	14.65(8.78,34.10)	16.30(12.50,24.83)	8.61 ± 3.21	<0.001	0.593
	DBIL(μmmol/l)	0-6.0	4.85(3.90,13.90)	6.50(4.83,10.28)	3.82 ± 1.36	<0.001	0.576
	IBIL(μmmol/l)	0-11.1	10.00(4.58,20.20)	10.40(8.15,15.20)	4.79 ± 1.96	<0.001	0.684
	ALB(g/L)	40.0-55.0	39.58 ± 6.76	36.69 ± 6.11	46.81 ± 3.85	<0.001	0.282
	GLO(g/L)	20.0-40.0	28.68 ± 5.34	34.72 ± 7.70	32.38 ± 3.38	0.218	0.054
	TBA(μmmol/l)	0-10.0	29.40(5.38,54.75)	30.60(13.53,52.43)	3.80 ± 0.96	<0.001	0.666
	CREA(μmmol/l)	44.0-133.0	72.00(60.05,88.75)	74.30(53.10,87.35)	57.13 ± 9.36	0.006	0.819
	BUN(mmol/L)	2.90-7.14	4.75(3.85,5.78)	4.82(3.94,7.26)	4.52 ± 1.00	0.241	0.576
	Na(mmol/L)	137.0-147.0	140.16 ± 2.80	140.47 ± 2.84	141.88 ± 2.19	0.072	0.800
	PT(s)	10.0-14.0	14.75 ± 2.68	14.59 ± 1.91	11.78 ± 0.80	<0.001	0.856
	PTA(%)	80.0-120.0	86.00(63.43,103.90)	71.35(68.00,80.10)	100.06 ± 10.32	<0.001	0.525
	INR	0.85-1.20	1.19 ± 0.25	1.23 ± 0.13	1.02 ± 0.09	<0.001	0.545

CPC, compensated cirrhosis; DCPC, decompensated cirrhosis; HC, healthy control; WBC, white blood cell count; N, neutrophil count; N%, neutrophil count over white blood cell count; LYM, lymphocyte count; Hb, hemoglobin; PLT, platelet count; ALT, alanine aminotransferase; AST, aspartate aminotransferase; TBIL, total bilirubin; DBIL, direct bilirubin; IBIL, indirect bilirubin; ALB, blood albumin; GLO, globulin; TBA, bile acid; CREA, creatinine; BUN, urea nitrogen; Na, serum sodium ion concentration; PT, prothrombin time; PTA, prothrombin time percentage; INR, international normalized ratio. * For comparison between CPC and DCPC (significant difference between groups, *p*<0.05).

#### 3.1.2 Microbial diversity between groups

**Figure 1:**
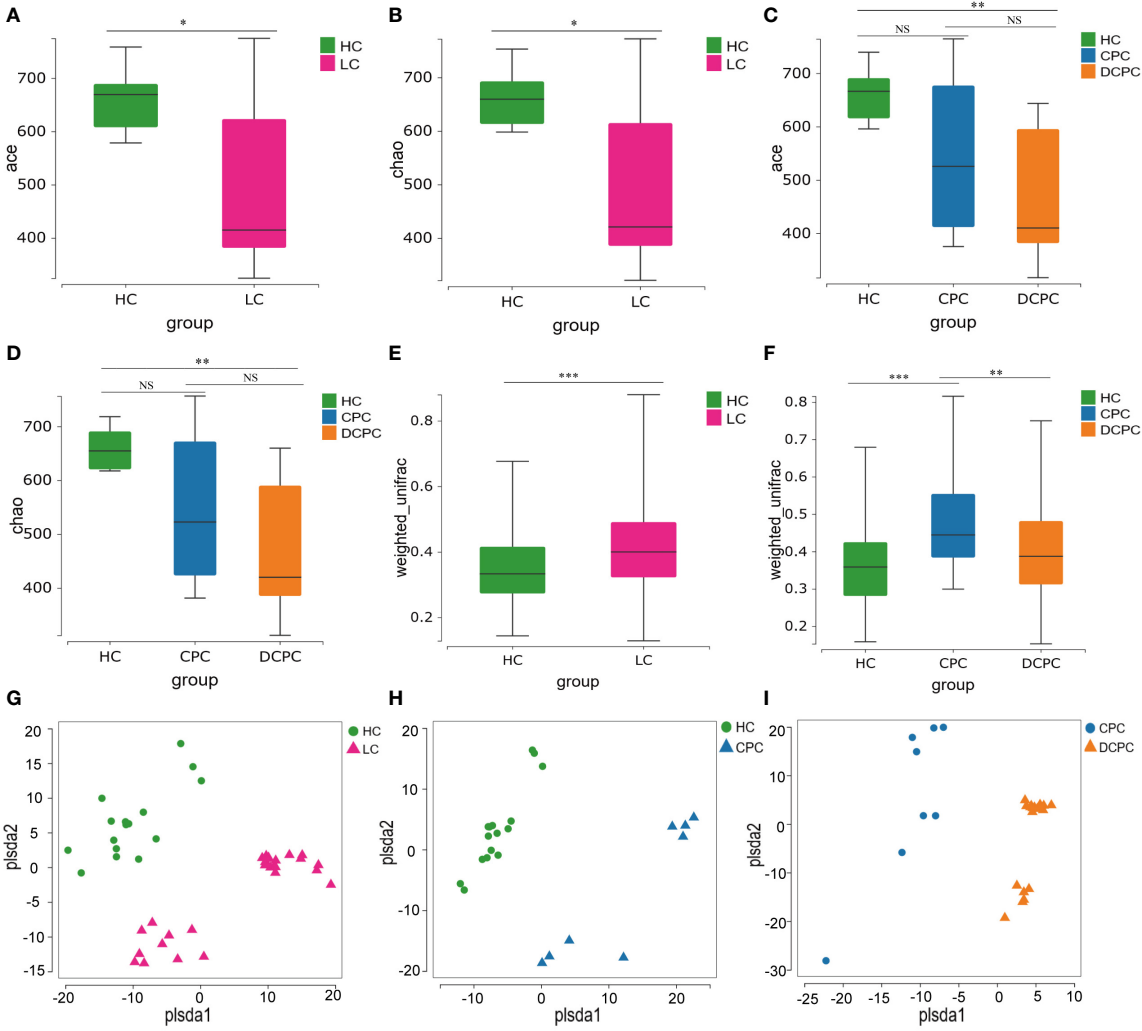
α and β diversity of gut microbiota between patients with liver cirrhosis and healthy controls presented by box plot and PLS-DA. α diversity was illustrated by the **(A, C)** Ace and **(B, D)** Chao1 indices. The β diversity was illustrated by the weight_UniFrac distance matrix-based method **(E, F)**. **(G)** PLS-DA between HC and LC; **(H)** PLS-DA between HC and CPC; **(I)** PLS-DA between CPC and DCPC. LC, liver cirrhosis; CPC, compensated cirrhosis; DCPC, decompensated cirrhosis. *p<0.05; **p<0.01; ***p<0.001. NS, not significant.

From fecal 16S rRNA sequencing, we first analyzed the gut microbiota profile in different groups and found that the α diversity of the gut microbiota, including the ACE and Chao1 index, was significantly reduced in patients with liver cirrhosis (LC) compared with healthy controls (HCs) (*p*<0.05) (**Figures 1A, B**). The gut microbiota profile in the decompensated cirrhosis group (DCPC) showed the lowest α diversity evaluated by the ACE and Chao1 index when compared with the HC group (**Figures 1C, D**). However, no significant changes in α diversity were observed in the comparison between the DCPC and CPC groups (**Figures 1C, D**).

Then, we analyzed the β diversity in patients with cirrhosis compared to healthy controls. Two independent fecal samples were detected, and clearly different clusters were observed in the cirrhosis and HC groups (**Figures 1E, F**). Additionally, distinct fecal microbiota profiles were also shown between CPC and HC, CPC and DCPC by using PLS-DA, indicating that the composition of gut microbiota was greatly different between cirrhosis and healthy subjects (**Figures 1G–I**).

#### 3.1.3 Differences in the composition of the gut microbiota between groups

**Figure 2:**
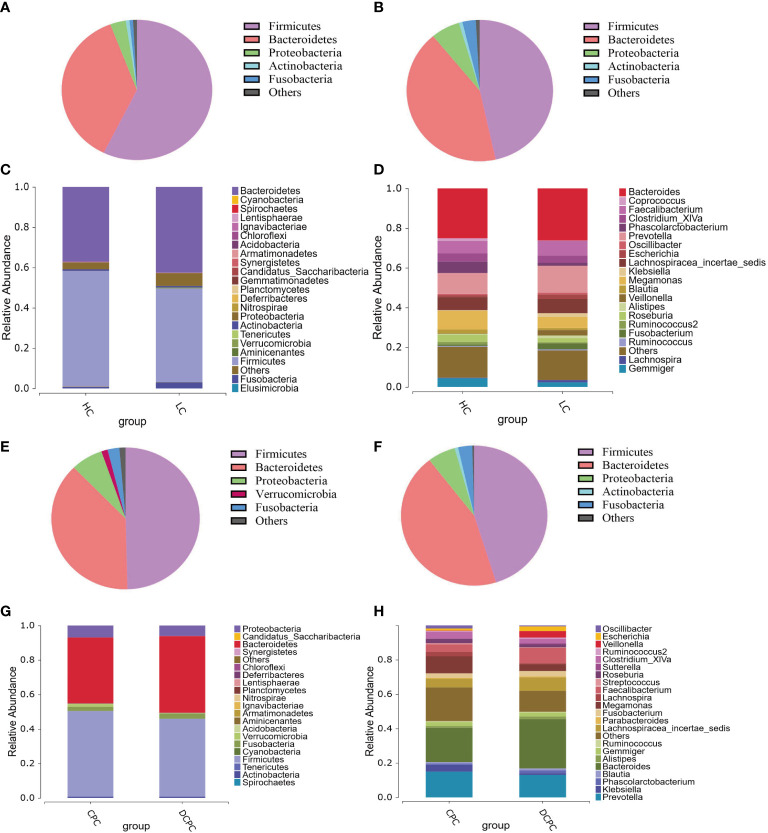
Pie charts and histograms of the gut microbiota composition in patients with liver cirrhosis and healthy controls. Pie charts were used to show the gut microbiota composition at the phylum level of healthy controls **(A)**, cirrhotic patients **(B)**, patients with compensated cirrhosis **(E)** and patients with decompensated cirrhosis **(F)**. Histograms were used to show the gut microbiota composition at the phylum level **(C, G)** and genus level **(D, H)** in cirrhotic patients and healthy controls.

Sequencing results showed an alteration in bacterial populations at the phylum level in the cirrhosis group compared with the HC group: *Firmicutes* (46.51% vs. 57.29%), *Bacteroidetes* (42.49% vs. 36.94%), *Proteobacteria* (6.36% vs. 3.36%), *Fusobacteria* (2.97% vs. 0.77%), and *Actinobacteria* (0.79% vs. 0.80%) (**Figures 2A–C**). The proportion of *Firmicutes* and *Bacteroidetes* in total bacteria was lower in patients with cirrhosis than in healthy individuals (88% vs. 94%). At the genus level, the predominant microbiota in the cirrhosis group compared with the HC group was *Bacteroides* (26.21% vs. 25.11%), *Prevotella* (13.51% vs. 10.53%), *Megamonas* (5.68% vs. 9.53%), and *Faecalibacterium* (7.54% vs. 6.32%) (**Figure 2D**).

More importantly, in patients with cirrhosis, the SCFA-producing species *Firmicutes*, *Lachnospiraceae*, *Ruminococcaceae*, *Coprococcus*, *Phascolarctobacterium*, and *Roseburia* were reduced compared to those in healthy subjects, with a significant reduction in *Firmicutes* and *Coprococcus* (*p* < 0.05). Moreover, since a decreased *Firmicutes*/*Bacteroidetes* ratio is generally seen in dysbiosis, particularly in inflammatory bowel disease (IBD)(Stojanov and Berlec, 2020), in our study, we found that the F/B ratio was decreased in cirrhotic patients versus healthy individuals (1.21 vs. 1.78), indicating that gut dysbacteriosis were existed in cirrhosis. Therefore, these results demonstrate that the levels of the beneficial bacteria *Firmicutes* and SCFA-producing bacteria in cirrhosis were significantly reduced compared to those in healthy subjects.

Additionally, in patients with compensated and decompensated cirrhosis, the composition of the gut microbiota at the phylum and genus levels was distinct, with a lower abundance of *Firmicutes* in the DCPC group than in the CPC group (**Figures 2E–H**). Lower levels of SCFA-producing bacteria, including *Lachnospiraceae*, *Ruminococcaceae*, and *Roseburia*, were also observed in patients with DCPC. In addition, the F/B ratio was also lower in patients with DCPC than in patients with CPC (1.04 vs. 1.38). We then used the modified dysbiosis ratio (MDR), which refers to (*Bacillus* class% + *Proteobacteria* phylum %)/(*Clostridium* class % + *Bacteroidetes* phylum %) (Maslennikov et al., 2021), to estimate microbial dysbiosis in patients with cirrhosis. The results indicated that MDR in the DCPC group was significantly higher than that in the CPC group (*p*<0.05), suggesting that microbial dysbiosis is severe when cirrhosis reaches the decompensated stage.

#### 3.1.4 Linear discriminant analysis effect size

**Figure 3:**
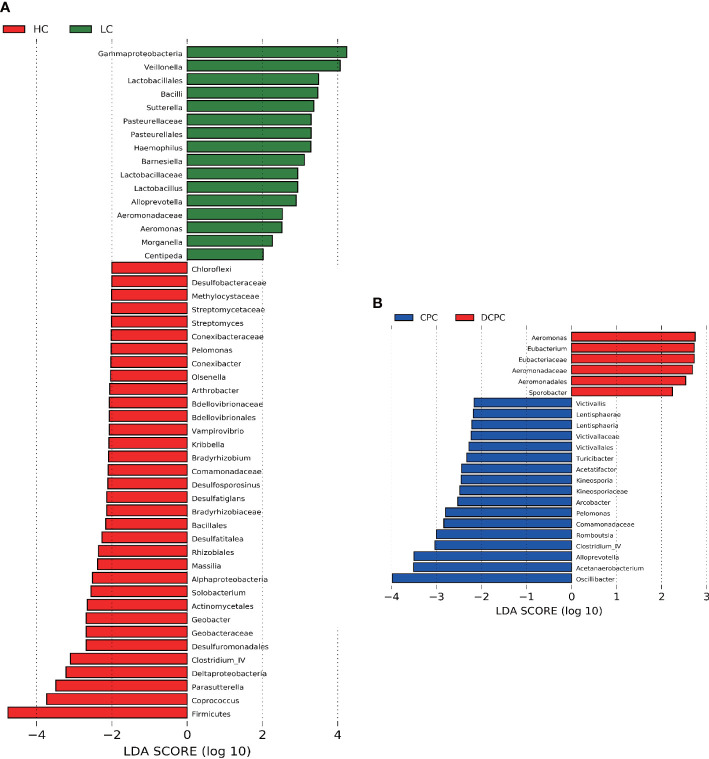
Difference in the gut microbiota in **(A)** LC and HC, **(B)** CPC and DCPC. Species with LDA scores greater than 2 are shown; the color of the bar represents the respective group, while the length represents the LDA score. LDA, linear discriminant analysis; LC, liver cirrhosis; CPC, compensated cirrhosis; DCPC, decompensated cirrhosis.

Differential analysis of gut microbiota between liver cirrhosis and healthy controls was used by LEfSe (linear discriminant analysis effect size), and we found that levels of *Firmicutes*, *Coprococcus*, *Parasutterella*, *Deltaproteobacteria* and *Clostridium IV* were significantly reduced in cirrhotic patients compared with healthy individuals. Notably, *Firmicutes*, *Coprococcus* and *Clostridium IV* are representative SCFA-producing bacteria. *Gammaproteobacteria*, *Veillonella*, *Lactobacillales*, and *Bacilli* were greatly enriched in cirrhotic patients (**Figure 3A**). In the comparison between the DCPC and CPC groups, the results showed that *Oscillibacter*, which is a SCFA-producing bacterium, was significantly reduced in DCPC, indicating that with the progression of cirrhosis, especially when reaching DCPC, microbial dysbiosis gradually deteriorated and presented with significantly reduced levels of SCFA-producing bacteria (**Figure 3B**).

#### 3.1.5 Levels of SCFAs were reduced in patients with liver cirrhosis

**Figure 4:**
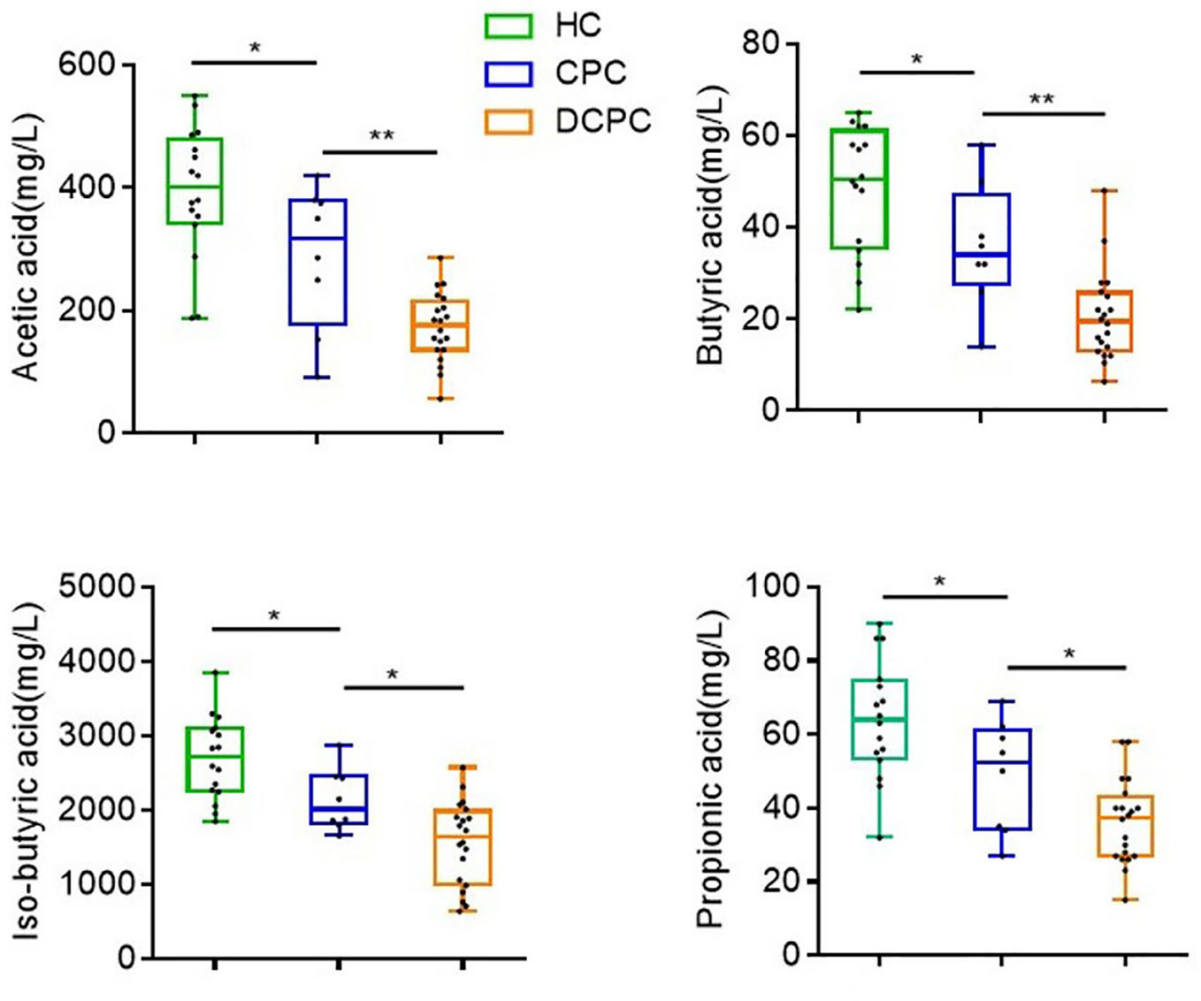
Differences in the levels of SCFAs in fecal samples among the groups. *p<0.05; **p<0.01.

Based on decreased levels of SCFA-producing species in cirrhosis patients, we further tested the levels of fecal SCFAs (acetic, butyric, isobutyric, and propionic acid) in cirrhotic patients and healthy individuals via HPLC. We found that the levels of acetic, butyric, isobutyric, and propionic acid were lowest in DCPC patients (173 ± 12.6; 20.6 ± 2.2; 1560 ± 126.4; 36.2 ± 2.5,respectively) when compared to CPC group (288.3 ± 41.3; 35.8 ± 4.8; 2136 ± 148.2; 48.9 ± 5.4) and healthy controls (393.7 ± 26.82; 48.6 ± 3.4; 2696 ± 139.5; 64 ± 3.9) (**Figure 4**).

#### 3.1.6 Functional categories were different between groups

To compare the functional genetic differences of the colonies, the 16S rRNA gene sequencing results were compared by Kyoto Encyclopedia of Genes and Genomes. The genes of gut microbiota in both healthy individuals and cirrhotic patients are extensively involved in metabolic activities, including carbohydrate, vitamin, amino acid, lipid, polysaccharide and nucleotide metabolism. Dietary fibers are degraded by intestinal SCFA-producing microbiota to produce SCFAs (including acetate, propionate and butyrate), and most gut bacteria can produce acetate, while propionate and butyrate are generally produced by specific microbiota(Martin-Gallausiaux et al., 2021). A variety of substrates, including amino acids, carbohydrates, and lactic acid, are needed in the production of propionate and butyrate (Martin-Gallausiaux et al., 2021).

**Figure 5:**
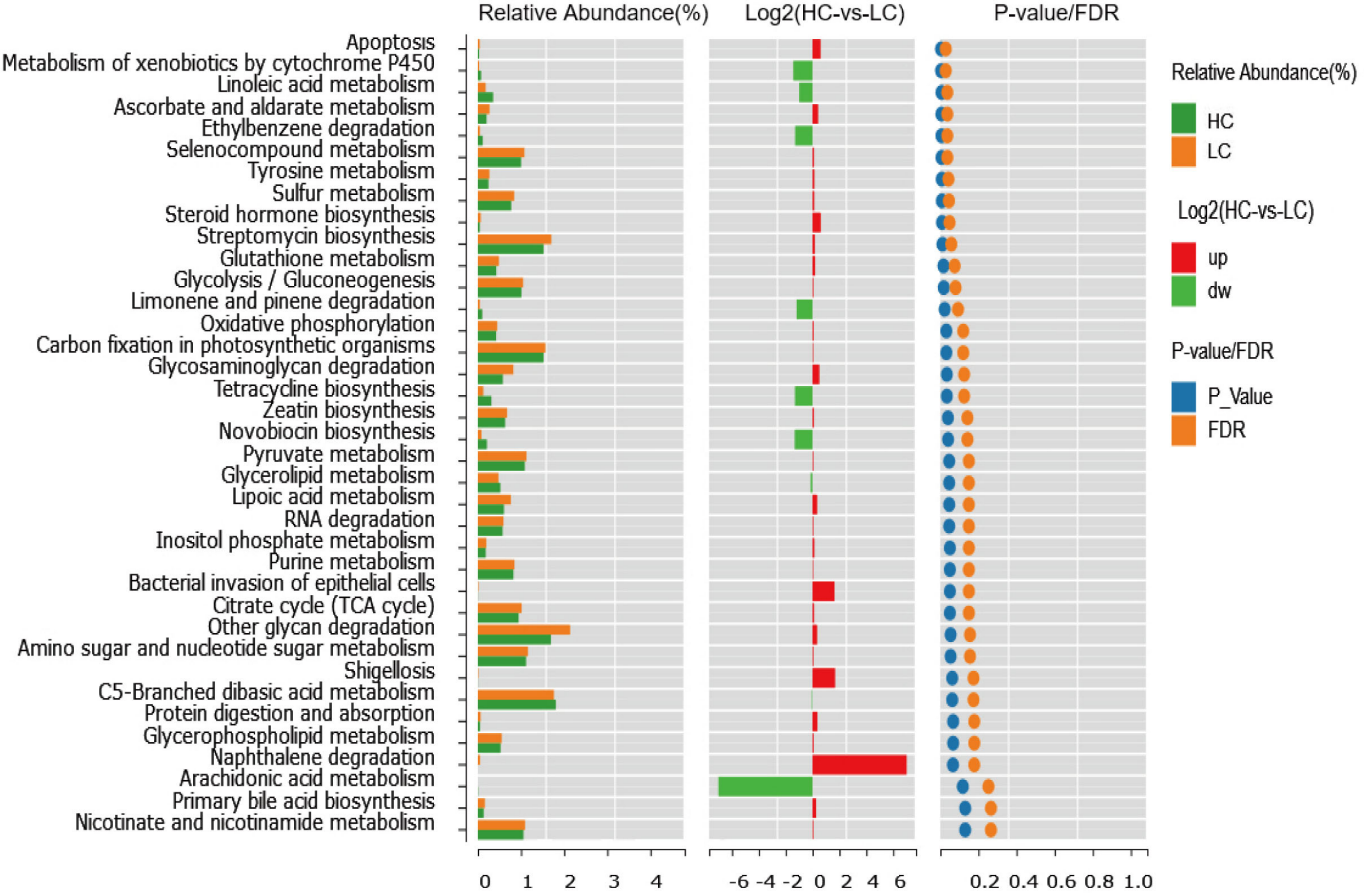
Differences in functional modules of the gut microbiota in LC and HC.

Overall, we found significant differences in functional modules in the comparison between cirrhosis patients and healthy individuals; a total of 165 functional modules were identified, among which 34 functional modules were found to be significantly different between healthy controls and patients with liver cirrhosis. Twenty-two functional modules were significantly enriched in cirrhotic patients, and 12 were more abundant in healthy controls. Pathways related to glycolysis/gluconeogenesis, peroxisome, taurine and hypotaurine metabolism, apoptosis, tyrosine metabolism, steroid hormone biosynthesis, glutathione metabolism and pyruvate metabolism were enriched in cirrhotic patients. In addition, pathways related to glycerolipid metabolism, polyketide sugar unit biosynthesis, metabolism of xenobiotics by cytochrome P_450_ and linoleic acid metabolism were diminished in cirrhotic patients. This result reveals that an altered gut microbiome brings changes in the functionality of the microbiome, which might contribute to the progression of cirrhosis (**Figure 5**).

#### 3.1.7 Correlation between gut microbiota profiles and hepatic function in cirrhosis

**Figure 6:**
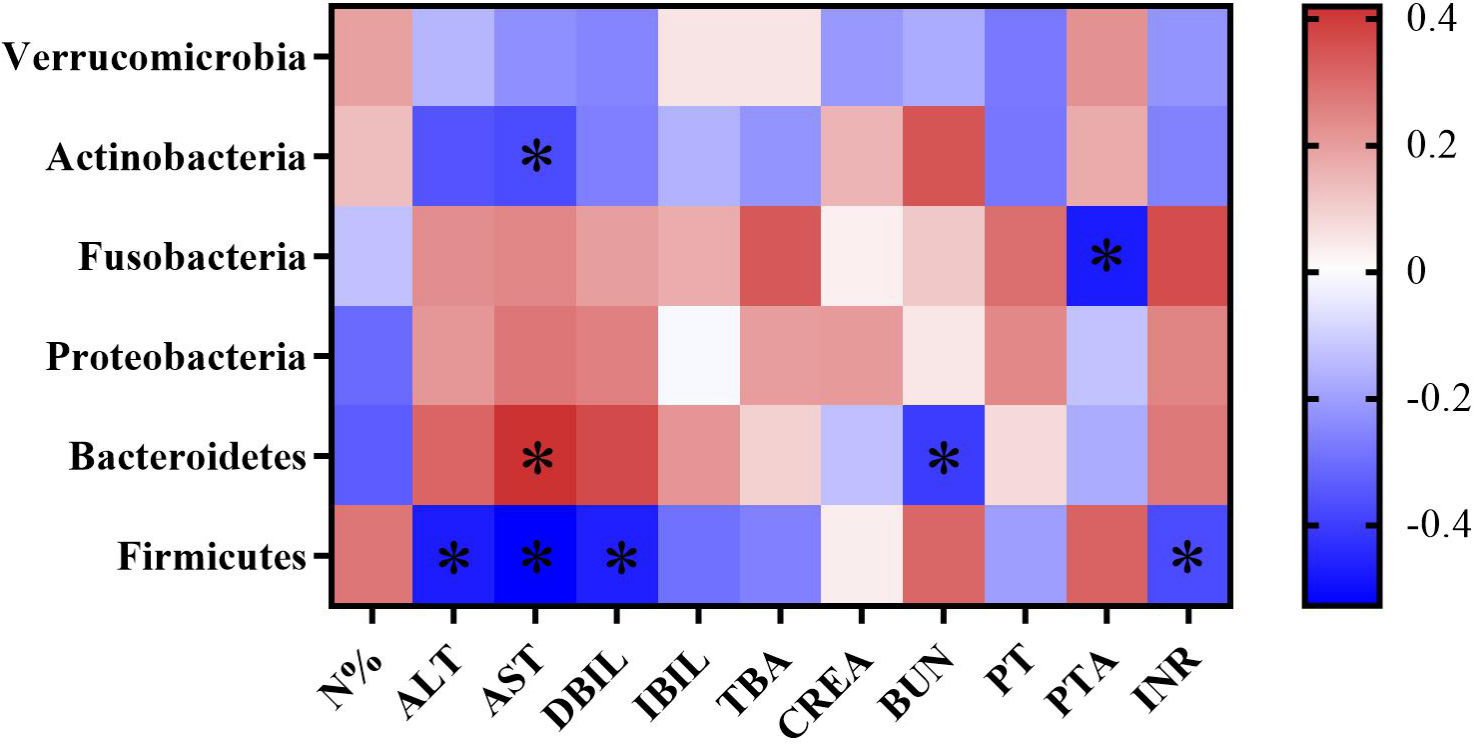
Heatmap of correlation coefficients between gut microbiota abundance and biochemical indicators in cirrhotic patients. Color and its depth represent the positive, negative and absolute magnitude of Spearman’s correlation coefficient r, respectively. *p < 0.05.

To evaluate the correlation between gut microbiota and the progression of cirrhosis, we correlated the abundance of gut microbiota with biochemical indicators such as liver function, kidney function and coagulation function. By Spearman’s correlation analysis, we found that at the phylum level, the abundance of *Firmicutes* was negatively correlated with ALT, AST, and DBIL levels (*p*<0.05), and the abundance of *Bacteroidetes* was positively correlated with AST level (*p*<0.05). Combined with the above finding that patients with cirrhosis had a greatly reduced abundance of *Firmicutes* compared to healthy individuals, we speculate that a decreased abundance of *Firmicutes* is closely associated with worsened liver dysfunction in cirrhotic patients (**Figure 6**).

### 3.2 Efficacy of adjuvant probiotic therapy in patients with cirrhosis

Then, as *Bifidobacterium* and *Lactobacillus* are important SCFA-producing bacteria (Lee et al., 2020; Lv and Liu, 2020), we used “Live Combined *Bifidobacterium*, *Lactobacillus*, and *Enterococcus* Capsules, Oral”, a commonly used probiotics in China to determine the role of supplementary SCFA-producing bacteria on the progression of cirrhosis. A total of 102 subjects who met the inclusion and exclusion criteria were included in this study and were divided into 58 cases in the nonprobiotics group and 44 cases in the probiotics group. In the nonprobiotic group, there were 40 males and 18 females, with a mean age of 51.52 ± 13.50 years. In the probiotics group, there were 37 males and 7 females, with a mean age of 55.00 ± 12.74 years. There was no significant difference in sex, age, Child-Pugh classification, pathogenesis or blood tests at the time of admission between the two groups (all *p >*0.05). The hematological parameters and hepatic profile were comparable between the probiotic and nonprobiotic groups and pre- and post-treatment in each group. We found that both the probiotic and nonprobiotic groups post-treatment had significantly lower levels of neutrophil ratio, alanine transaminase, aspartate aminotransferase, total bilirubin and total bile acid compared with their pretreatment, respectively (*p* < 0.05), and levels of alanine aminotransferase and total bilirubin were markedly lower in probiotic group post-treatment than nonprobiotic group post-treatment (*p* < 0.05). Therefore, in patients with cirrhosis, the use of probiotics especially related to SCFA production in addition to standard treatment can be helpful in improving the patients’ hepatic function (**Table 2**).

**Table 2:** Demographic information and clinical data of patients in both the probiotic and nonprobiotic groups.

	Probiotic group		Nonprobiotic group	
Gender				
Male	37		40	
Female	7		18	
**Age**	55.00 ± 12.74		51.52 ± 13.50	
Pathogenesis				
Hepatitis	17		26	
Alcoholic	10		9	
Mixed Pathogenesis	8		8	
unclassified	9		15	
Child-pugh grade				
A	4		16	
B	25		28	
C	15		14	
	Pretreatment	Post-treatment	Pretreatment	Post-treatment
WBC	3.52(2.71-4.52)	3.57(2.49-4.60)	3.50(2.47-4.59)	3.35(2.48-4.35)
N%	63.03 ± 11.74	60.39 ± 11.19*	63.21 ± 13.21	57.29 ± 12.93*
Hb	110.8 ± 21.28	109.77 ± 21.86	103.81 ± 24.98	102.21 ± 24.22
PLT	66.50(41.00-99.75)	68(43.25-105.00)	64.00(45.75-108.00)	67.50(45.75-103.25)
ALT	31.85(26.55-45.95)	23.35(18.10-36.83)*+	39.90(25.58-55.98)	33.70(21.75-53.78)*
AST	52.00(33.83-76.55)	41.10(25.65-53.53)*	57.85(41.20-77.45)	46.35(36.18-66.28)*
TBIL	39.90(28.80-59.00)	27.80(17.75-46.78)*+	37.60(29.68-56.88)	34.90(26.08-49.13)*
TBA	49.40(23.98-94.75)	45.50(19.00-77.50)*	51.65(23.08-106.05)	44.60(24.83-76.83)*

*indicates a statistically significant difference between pre- and posttreatment (*p* < 0.05). +indicates a statistically significant difference between the probiotic and nonprobiotic groups posttreatment (*p* < 0.05).

## 4 Discussion

The gut microbiota and its metabolites can impact host digestion, metabolism and immunity and are critical in human health and disease progression. The gut-liver axis constructs the bidirectional connection between the gut and liver through the portal vein and biliary system. The gut microbiome is closely associated with the liver environment, which always acts as a biological initiator to trigger chronic inflammation and fibrosis upon microbial dysbiosis. In this study, we found that the gut microbiota profile in patients with cirrhosis was remarkably different from that of healthy individuals, presenting with significantly reduced α diversity of gut microbiota and decreased abundance of *Firmicutes*, particularly SCFA-producing microbiota. Furthermore, in a retrospective study, administration of probiotics to patients with cirrhosis effectively improved hepatic function compared to patients who received nonprobiotic treatment. Therefore, we concluded that gut microbial imbalance exists in patients with cirrhosis, and adjuvant probiotic therapy can be helpful in improving the liver function of patients with cirrhosis.

Gut microbial dysbiosis is generally present in liver cirrhosis, presenting with decreased richness of the gut microbiome, a reduction in autochthonous taxa, including *Lachnospiraceae*, *Ruminococcaceae*, and *Clostridiales XIV*, and an increase in pathogenic taxa such as *Enterococcaceae*, *Staphylococcaceae*, and *Enterobacteriaceae*, as previously reported (Bajaj et al., 2014; Bajaj et al., 2015; Chen et al., 2011). In our study, we found that the α diversity was significantly reduced in LC patients with a distinct composition of the gut microbiome, manifesting with a decreased abundance of *Firmicutes* and an increased level of *Bacteroidetes* at the phylum level. At the genus level, *Coprococcus and Clostridium IV* were significantly reduced, while *Veillonella* was enriched in LC. When comparing the CPC and DCPC groups, we found that *Firmicutes* and *Oscillibacter* were reduced in the DCPC group. Additionally, previous studies have mainly focused on alterations in α diversity and β diversity of gut microbiota in cirrhosis. Since an altered *Firmicutes* to *Bacteroidetes* ratio was correlated with microbial dysbiosis and patients with IBD showed a decreased F/B ratio (Stojanov and Berlec, 2020), we used F/B to assess microbial imbalance and found that the F/B ratio was higher in healthy individuals than in cirrhotic patients, and patients with CPC also had a higher F/B ratio than DCPC patients. In addition, patients with DCPC also had higher MDR values than CPC patients, indicating that severe bacterial imbalance was present in patients with cirrhosis, especially when reaching the decompensation stage.

SCFAs, mainly consisting of acetate, propionate and butyrate, can be produced by specific gut bacteria by fermentation of dietary fibers. SCFAs maintain gut integrity by regulating gut luminal pH, mucus production and mucosal immune function, and they also serve as an important energy source for epithelial cells (Morrison & Preston, 2016; Blaak et al., 2020). It has been shown that a decrease in gut SCFAs is associated with the progression of local or systematic diseases, such as inflammatory bowel disease (Parada Venegas et al., 2019), colon cancer(Hou et al., 2022), liver (Bajaj and Khoruts, 2020) and cardiovascular diseases (Hu et al., 2022). SCFAs can be absorbed in the colon and then transferred to the liver via the portal vein (Martin-Gallausiaux et al., 2021). In cirrhosis, the levels of SCFAs are markedly decreased (Baltazar-Díaz et al., 2022), and supplementary SCFAs may have regulatory effects in improving liver function. SCFAs can reduce liver inflammation by inhibiting M1 macrophages and increasing M2 macrophages, thereby resolving alcoholic liver disease (Wang et al., 2020). In addition, supplementation with SCFAs has emerged as a potential therapeutic approach in a variety of liver diseases because of its protective role in intestinal permeability (Pohl et al., 2022). In this study, when analyzing the levels of SCFA-producing bacteria between different groups, we found that *Firmicutes*, *Coprococcus* and *Clostridium IV* were significantly reduced in cirrhotic patients. The decreased abundances of *Firmicutes* and *Coprococcus* have been shown in cirrhosis as previously reported (Baltazar-Díaz et al., 2022; Li et al., 2022; Rodriguez-Diaz et al., 2022). *Clostridium IV* is a SCFA producer (Jin et al., 2022), which is an innovative finding in our study, and further investigations may focus on the relationship between *Clostridium IV* and cirrhosis. In addition, when compared with the species between DCPC and CPC patients, *Oscillibacter* was significantly reduced in the DCPC group. *Oscillibacter* is also a SCFA-producing bacterium (Jin et al., 2022) that is negatively linked to triglyceride concentration (Liu et al., 2022), and its level could facilitate the diagnosis of preeclampsia. Zhou et al. demonstrated that *Oscillibacter* was enriched in a long-term high-fructose diet mouse model and was associated with hepatic steatosis(Zhou et al., 2023). The role of *Oscillibacter* in the progression of liver cirrhosis requires further larger and deeper research. Therefore, the reduction in SCFA-producing bacteria in the gut microbiome of cirrhotic patients may bring decreased production of SCFAs, resulting in an impaired intestinal barrier and then triggering intestinal and liver inflammation, which is responsible for liver damage and cirrhosis. The species of SCFA-producing bacteria were even fewer in decompensated cirrhosis.

In our study, *Gammaproteobacteria* and *Veillonella* were enriched in cirrhotic patients. *Gammaproteobacteria* are related to many diseases, such as chronic intestinal infective disease (Salimi et al., 2022) and chronic kidney disease (Xu et al., 2017), and are therefore widely known as enteropathogenic bacteria. *Veillonella* is a lactic acid-fermenting bacterium normally present in the oral cavity (Loomba et al., 2021), and it is also a gut commensal bacterium (Shao et al., 2018). The existing findings support that increased richness of *Veillonella* is closely related to the progression of various liver diseases, including autoimmune hepatitis, primary biliary cirrhosis, and alcoholic hepatitis (Lv et al., 2016; Cortez et al., 2020; Lang et al., 2020; Kim et al., 2021; Wei et al., 2020), and it is also positively correlated with the level of alpha fetoprotein (AFP) in patients with primary liver cancer (Zhang et al., 2019). In cirrhosis, *Veillonella* was correlated with the severity of cirrhosis, and it was enriched in patients with acute onset overt hepatic encephalopathy (Sung et al., 2019). Our study found an increase in *Veillonella* in the gut microbiota of patients with cirrhosis, which is consistent with previous findings (Shao et al., 2018; Tang et al., 2021).

An altered gut microbiome may lead to altered functionality of the microbiome, which may be a key factor for the induction of intestinal inflammation, disruption of the intestinal barrier, and translocation of microbial material, thus aggravating liver inflammation and intestinal dysbiosis, which may contribute to the progression of cirrhosis. Glycolysis can produce pyruvate, and pyruvate is fermented in an anaerobic environment to produce lactate; the lactate level is increased in cirrhotic patients (Scheiman et al., 2019). In addition, a high lactate level is related to a worse prognosis of hepatitis B virus-related decompensated cirrhosis (Nie et al., 2021). These previous studies support our finding that functional modules related to glycolysis and pyruvate metabolism were significantly increased in cirrhotic patients in this study. Notably, SCFAs, which are highly relevant to the development of lipid accumulation, presented with enhanced glycerolipid metabolism and the PPAR signaling pathway (Chang et al., 2023). In addition, linoleic acid is related to SCFA production, and supplementation with linoleic acid in mice can enhance the levels of SCFAs, including cecal acetate, propionate and isobutyrate(Marques et al., 2015). In our study, pathways related to glycerolipid metabolism and linoleic acid metabolism were greatly diminished in cirrhotic patients, which might suggest reduced levels of SCFAs in cirrhosis. Therefore, alterations in the gut microbiota cause changes in functional modules, which might indicate the decreased production of SCFAs in cirrhosis, contributing to disease progression.

Worsen microbial dysbiosis occurred with the progression of cirrhosis and was associated with poor prognostic outcomes. Patients with acute-on chronic liver failure (ACLF) had enriched Enterococcus and lower microbial richness than DCPC patients without ACLF, indicating the relationship between microbial dysbiosis and hepatic complications and disease severity (Solé et al., 2021). Due to the important regulatory role of the gut microbiota in hepatic function, in our study, we found that the abundance of *Firmicutes* was negatively correlated with the levels of ALT, AST, and DBIL. Based on the reduced richness of *Firmicutes* in cirrhotic patients, particularly in DCPC patients in our study, we demonstrated that a reduction in *Firmicutes* in cirrhotic patients might be associated with worse hepatic function and poor prognosis. In addition, the abundance of *Fusobacteria* was negatively correlated with the PTA level. Although the abundance of *Fusobacteria* in the microbiota profile of patients with cirrhosis was higher than that in healthy individuals, the results were not significant, which reveals that the abundance of *Fusobacteria* may be related to hepatic function and facilitate the assessment of the severity of cirrhosis. Further studies are needed to confirm this finding.

Because the gut microbiota of cirrhotic patients was markedly altered and this change was associated with the progression of cirrhosis, we then conducted a retrospective study to explore the role of probiotics in liver cirrhosis. Since probiotics do not have a significant beneficial effect on patients with compensated cirrhosis (Pereg et al., 2011), we explored the therapeutic effect of oral probiotic supplementation in patients with DCPC. We found that oral probiotics could effectively improve liver function in patients with cirrhosis, such as alanine aminotransferase (ALT) and total bilirubin (TBIL). A previous study reported the role of microbial therapy in reducing ALT levels in patients with nonalcoholic fatty liver disease (Loman et al., 2018). In addition, probiotic supplementation significantly reduced ALT levels and benefited liver injury in an animal model of acute liver injury and liver cirrhosis (Adawi et al., 2001; Liu et al., 2015; Liu et al., 2017; Zhang et al., 2022). In addition, treatment with probiotics prior to liver transplantation in cirrhotic patients was associated with improvements in early postoperative ALT (Grąt et al., 2017). Additionally, a modified gut microbiota after probiotic treatment results in reduced bilirubin in acute liver injury (Xu et al., 2021). In this study, oral intake of probiotics also resulted in decreased TBIL levels in cirrhotic patients, which was also consistent with a previous study (Chen et al., 2017). Therefore, probiotic therapy especially supplementing with SCFA-producing bacteria has been shown to improve liver function in patients with cirrhosis via alteration of microbiota dependent on the gut-liver axis.

In our study, we excluded patients who received antibiotic treatment, which will eliminate the impact of antibiotics on gut microbiota and could reflect more accurate changes in gut microbiota in patients with cirrhosis than a previous study (Solé et al., 2021). This also explains why fewer Child-Pugh C or hepatic failure patients were included in this study, as these patients may need antibiotic therapy after admission. It can also explain why there is no significance of α diversity between the DCPC and CPC groups. In addition, we conducted a retrospective study to identify the effect of gut microbiota alteration on hepatic function in addition to fecal analysis of the gut microbiome and its correlation with disease outcome. However, some issues important to the interpretation of the current findings deserve discussion. First, a greater number of LC patients with different pathogeneses and healthy controls should be enrolled, and multicenter studies with larger sample sizes are also needed. Second, the use of 16S rRNA sequencing limited further analysis of microbial composition and function, and it cannot cover all species with 100% coverage, therefore metagenomics sequencing (MGS) is needed in future studies. Third, we only collected stool samples that could not fully represent mucosal microbiota, and further serum and mucosal samples can be collected.

In summary, we concluded that LC patients have altered gut microbiota, presenting with reduced microbial richness, enriched pathogenic Veillonella bacteria, and reduced SCFA-producing bacteria, including Firmicutes, Coprococcus, and Clostridium IV. The altered gut microbiome leads to changes in the functionality of the microbiome, which might contribute to the progression of cirrhosis. Moreover, Firmicutes was negatively correlated with various liver indicators, and an increased level of Firmicutes may predict a good prognostic outcome. Furthermore, DCPC patients who received probiotics had significantly improved hepatic function compared to the nonprobiotic group, and the use of adjuvant probiotic therapy to supply with SCFA-producing bacteria can be helpful in improving the hepatic function and prognosis of patients with cirrhosis.

## Data availability statement

The original sequencing data in the study are publicly available. Our data will be accessible with the following link after the indicated release date or on publication: https://www.ncbi.nlm.nih.gov/sra/PRJNA976251.

## Ethics statement

The studies involving human participants were reviewed and approved by the Ethics Committee of the Second Xiangya Hospital of Central South University (number: LYF2022112). The patients/participants provided their written informed consent to participate in this study. Written informed consent was obtained from the individual(s) for the publication of any potentially identifiable images or data included in this article.

## Author contributions

ZW: manuscript draft, data acquisition and analysis. HZ: material and technological support. DL: manuscript revision, study design and supervision. FD: study concept and design, obtained funding and study supervision. All authors contributed to the article and approved the submitted version.
